# Supplementary material for: The transcribed pseudogene RPSAP52 enhances the oncofetal HMGA2-IGF2BP2-RAS axis through LIN28B-dependent and independent let-7 inhibition
Source: Nat Commun. 2019 Sep 4;10:3979. doi: 10.1038/s41467-019-11910-6 (PMC6726650; doi:10.1038/s41467-019-11910-6)
Supplement: Supplementary file 1 — Supplementary Information [file 41467_2019_11910_MOESM1_ESM.pdf]

Supplementary Information

The transcribed pseudogene *RPSAP52* enhances the oncofetal HMGA2-IGF2BP2-RAS axis through LIN28B-dependent and independent *let-7* inhibition

Oliveira-Mateos et al

This document contains:

- **Supplementary Figures 1-7.**
- **Supplementary Table 1. Oligos used in this work.**

a

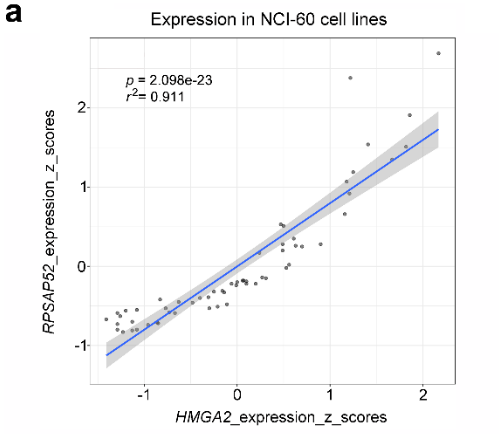

b

Alternative exon (- strand)
5'AACTTGGGTGCTACCACTTGGATCCTGAGATGCAAGTCAAGTAGCAGAGAGCAAC
GAGCTGGCCAGAGCCTGGGTCTGTGGTATTATGGAGCACCACACTGGAG 3'

c

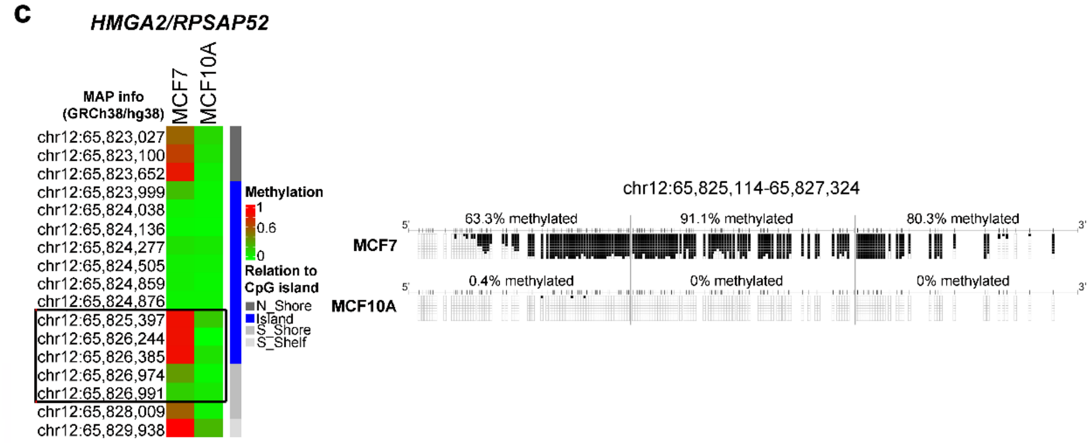

Supplementary Fig. 1. Expression and methylation of the *HMGA2/RPSAP52 locus*. **a** Correlation between *HMGA2* and *RPSAP52* in the NCI60 panel of cancer cell lines. Normalized expression array data (Z-score) are represented. R squared of Pearson correlation coefficient is shown. **b** The 104 nucleotides of *RPSAP52* alternative exon are indicated. **c** *Left*, heatmap representing methylation levels in MCF7 and MCF10A cell lines. The black square indicates the DNA region that was subject to sequencing following bisulfite treatment (*right*). 3 overlapping DNA fragments were analyzed, so that every CpG is interrogated between coordinates chr12:65,825,114 and chr12:65,827,324 (hg38). Vertical lines represent CpG positions along the whole sequence. Individual clones sequenced are represented horizontally, with empty squares corresponding to unmethylated CpGs and filled squares corresponding to methylated positions. Average methylation levels for each fragment are indicated. Source data for **c** are in Oliveira-Mateos et al_Source Data 1.

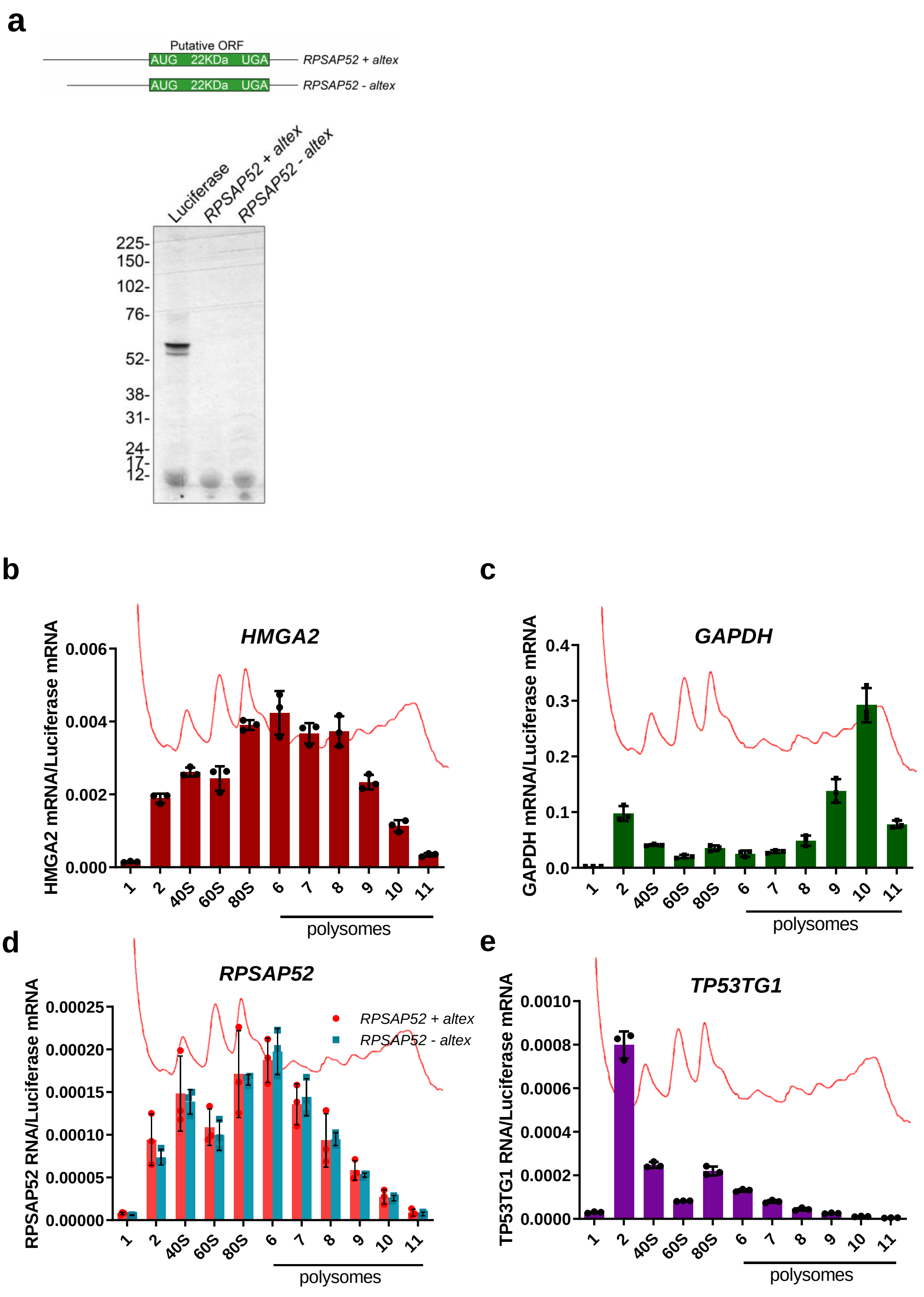

Supplementary Fig. 2. *RPSAP52* transcripts are associated with polysomes in MCF10A cells. a Transcription/Translation assay to test the coding potential of *RPSAP52*. A DNA fragment corresponding to the Luciferase open reading frame was used as positive control. The transcripts corresponding to *RPSAP52* + *altex* and *RPSAP52* – *altex* isoforms were assayed (both have a predicted encoded protein of 22 kDa, as

indicated in the upper diagram). The molecular weight for Luciferase is 61 kDa. **b-e** Polysome distribution on a 10%-50% sucrose gradient from MCF10A wild-type cells. The presence of the indicated transcripts in each fraction was analyzed by RT-qPCR. *GAPDH* and *TP53TG1* were used for comparison since they represent an actively translated and a non-protein coding transcript, respectively. Data are means ±SD, and error bars represent 3 replicates of RT-qPCR from each fraction. The red line indicates absorbance at 260 nm for each fraction. Source data for **b-e** are in Oliveira-Mateos et al_Source Data 1. Unprocessed scans are available in Oliveira-Mateos et al_Source Data 2.

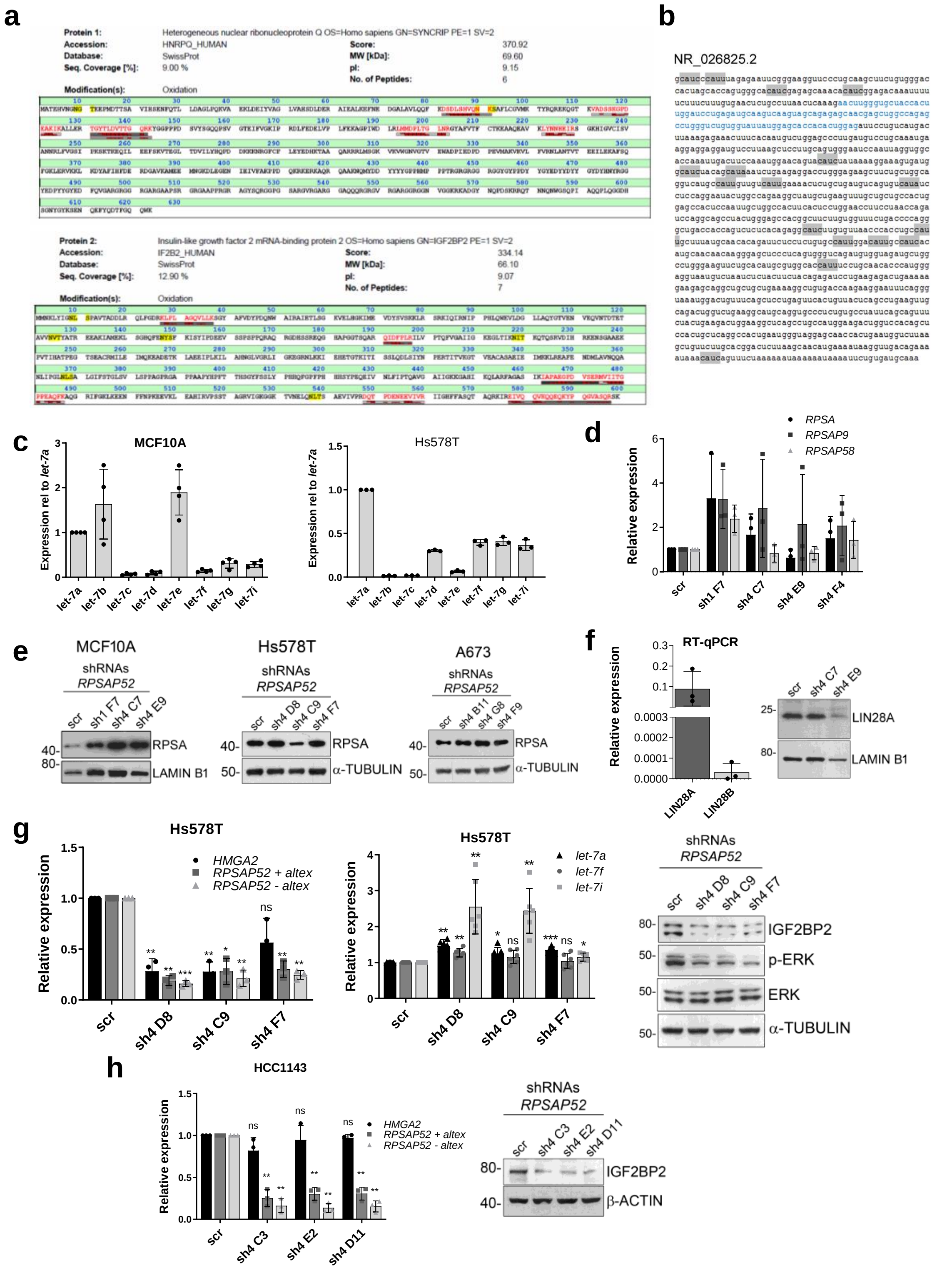

Supplementary Fig. 3. *RPSAP52* loss impacts on IGF2BP2/*let-7* pathway in breast cancer cell lines. a Mass spectrometry (MS) identification of the band isolated in the *RPSAP52* RNA pull-down. The specific band was cut out from the gel and trypsin digested for MS analysis, which detected 6 peptides for HNRNPQ protein and 7 peptides for IGF2BP2 protein. **b** *RPSAP52* pseudogene contains several CAU(H) motifs (shaded

in grey). The sequence of the alternative exon is highlighted in blue. **c** RT-qPCR analysis of the expression of *let-7* family members in MCF10A cells (left) and Hs578T (right). Abundance is expressed relative to *let-7a*. Data are means of at least three independent RNA extractions ±SD. **d** Expression of the *RPSA* mRNA and the pseudogenes *RPSAP9* and *RPSAP58* upon knockdown of *RPSAP52*. Total RNA from MCF10A clones stably expressing shRNAs against *RPSAP52* was analyzed by RT-qPCR. Data are means of three independent RNA extractions ±SD. **e** Western Blot to analyze RPSA levels upon *RPSAP52* depletion in MCF10A, Hs578T and A673 cell lines. **f** *LIN28A/B* expression in MCF10A cells. *Left*, mRNA relative expression as measured by RT-qPCR from 3 different RNA extractions. Data are means ±SD; *right*, Western Blot of LIN28A protein. LIN28B protein is undetectable in MCF10A cells. **g** RNA and protein analysis of Hs578T clones stably expressing shRNA4 against *RPSAP52. Left*, RT-qPCR analysis of *HMGA2* and *RPSAP52* expression. Three different total RNA extractions were analyzed, and two-tailed student *t*-tests were used (**P*<0.05, ***P*<0.01, ****P*<0.001, ns=not significant). Data are means ±SD. *Middle,* RT-qPCR to assess *let-7* miRNAs levels. Six RT-qPCR analysis were performed from three different total RNA extractions, and two-tailed student *t*-tests were used (**P*<0.05, ***P*<0.01, ****P*<0.001, ns=not significant). Data are means ±SD. *Right* (*image*), Western Blot to analyze IGF2BP2, total ERK and phosphorylated ERK (p-ERK) protein levels in the same clones. **h** RNA and protein analysis of HCC1143 clones stably expressing shRNA4 against *RPSAP52. Left*, RT-qPCR analysis of *HMGA2* and *RPSAP52* expression. Three different total RNA extractions were analyzed, and two-tailed student *t*-tests were used (**P*<0.05, ***P*<0.01, ns=not significant). Data are means ±SD. *Right* (*image*), Western Blot to analyze IGF2BP2 protein levels. Source data for **c**, **d**, and **f-h** are in Oliveira-Mateos et al_Source Data 1. Unprocessed scans are available in Oliveira-Mateos et al_Source Data 2.

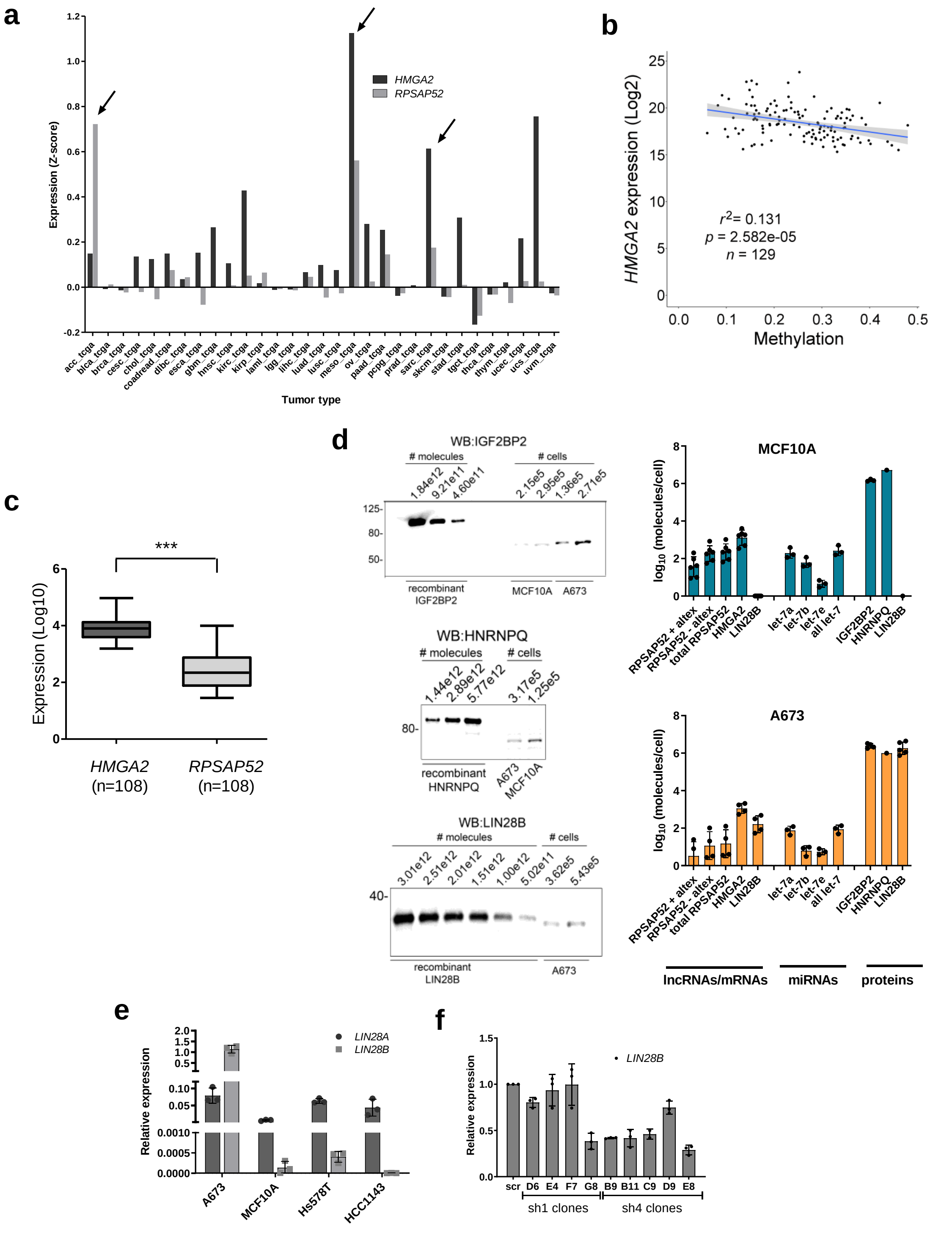

Supplementary Fig. 4. Evaluation of relative and absolute abundance of the RNAs and proteins involved in the regulatory network. a Z-score values for the expression of *HMGA2* and *RPSAP52* transcripts in all tumor types available at the TCGA database. Tumor types with the highest *RPSAP52* expression are indicated by an arrow. **b** *HMGA2* expression in the TCGA sarcoma cohort displays a weak negative correlation with the methylation of its associated CpG island, as measured by Pearson's coefficient. **c** Box

plots of *HMGA2* and *RPSAP52* relative expression levels in the TCGA cohort of sarcomas. Only patients with a recorded (non-zero) value for *RPSAP52* expression were considered. (****P*<0.001, two-tailed Mann-Whitney U test). The central mark of the box plot indicates the median, and the bottom and top edges of the box indicate the interquartile range (IQR). The box plot whiskers represent the minimum and maximum of all of the data. **d** Absolute estimations of number of transcripts and proteins per cell. Recombinant proteins or *in vitro* transcribed templates of known amounts were used for comparison. *Left images*, increasing amounts of each recombinant protein were loaded onto SDS/PAGE gels together with total extracts from an exact number of MCF10A or A673 cells, as indicated, and blotted with the corresponding antibodies. Densitometry analysis of the Western Blot signal was used for absolute estimation. *Right graphs*, the number of molecules per cell (log_{10}) in each cell line is shown for RNAs (*RPSAP52*, *HMGA2* and *LIN28B*), *let-7* miRNAs and proteins (IGF2BP2, HNRNPQ and LIN28B). Data are means ±SD, and error bars represent results from at least 2 different experiments (1 for HNRNPQ). **e** *LIN28A/B* mRNAs expression in the cell lines indicated. Relative expression was measured by RT-qPCR taking as a reference *LIN28B* levels in A673 cells. Data are means ±SD, and error bars represent 3 replicates of RT-qPCR from different RNA extractions. **f** RT-qPCR analysis of *LIN28B* mRNA levels upon *RPSAP52* knockdown in A673 cells. Data are means ±SD, and error bars represent 3 replicates of RT-qPCR. Source data for **d-f** are in Oliveira-Mateos et al_Source Data 1. Unprocessed scans are available in Oliveira-Mateos et al_Source Data 2.

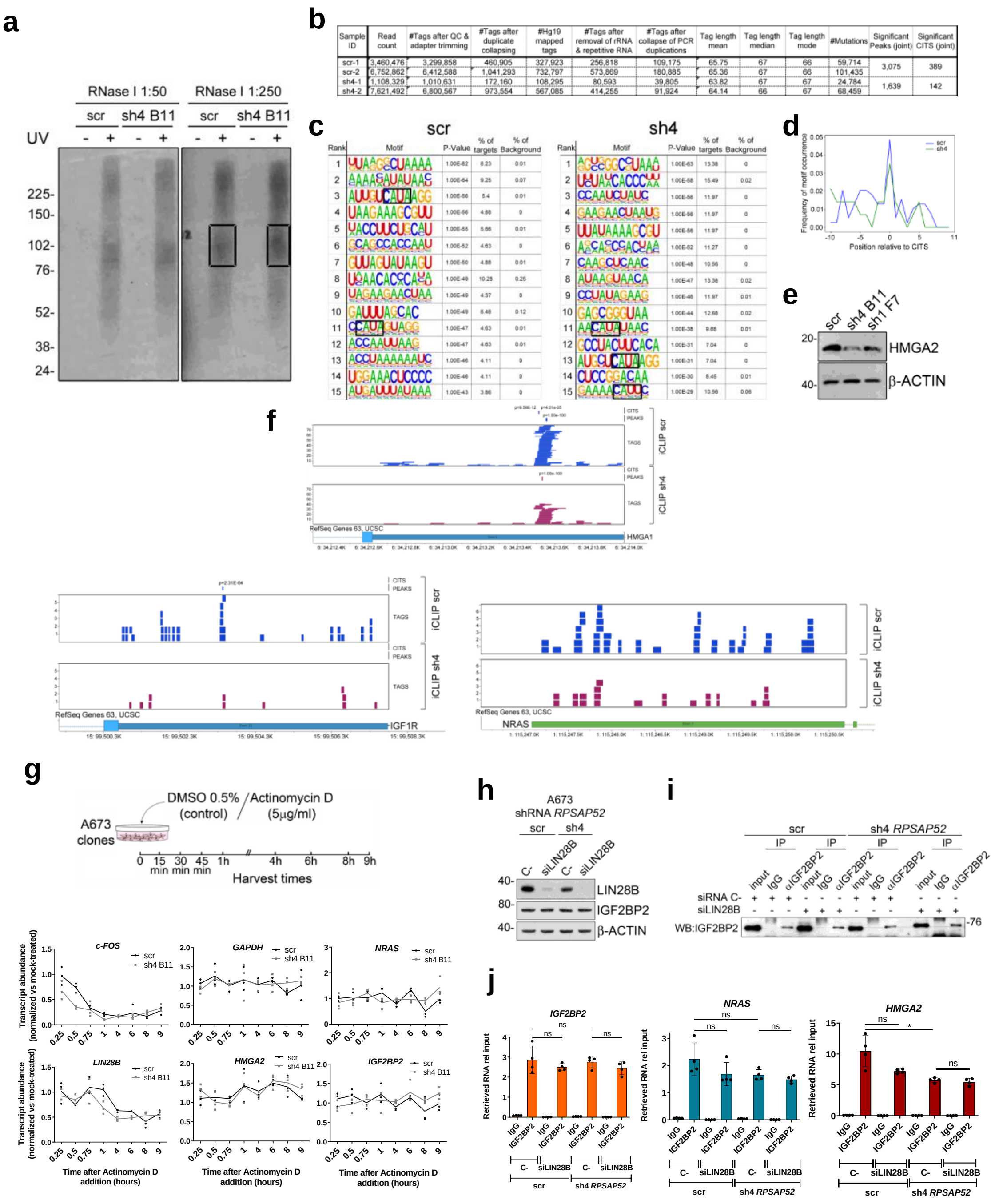

Sample ID	Read count	#Tags after QC & adapter trimming	#Tags after duplicate collapsing	#Hg19 mapped tags	#Tags after removal of rRNA & repetitive RNA	#Tags after collapse of PCR duplications	Tag length mean	Tag length median	Tag length mode	#Mutations	Significant Peaks (joint)	Significant CITS (joint)
scr-1	3,460,476	3,299,858	460,905	327,923	256,818	109,175	65.75	67	66	59,714	3,075	389
scr-2	6,752,862	6,412,588	1,041,293	732,797	573,869	180,885	65.36	67	66	101,435		
sh4-1	1,108,329	1,010,631	172,160	108,295	80,593	39,805	63.82	67	67	24,784	1,639	142
sh4-2	7,621,492	6,800,567	973,554	567,085	414,255	91,924	64.14	66	67	68,459		

Supplementary Fig. 5. iCLIP-seq experiment of IGF2BP2 in the context of *RPSAP52* depletion. a Autoradiograph of IGF2BP2 iCLIP experiment in A673 cells expressing control shRNAs (scr) or the sh4 sequence against *RPSAP52* (sh4 B11). Two different concentrations of RNaseI were tested, and for each condition a –UV control was included. The excised RNA-protein bands are marked by the black squares. **b** iCLIP-seq experiment statistics. Results for each of the two experimental replicates for each condition are indicated. **c** Sequence logos of the IGF2BP2 RNA binding motif for each condition, generated by Homer analysis of all significant CITS positions (+/-10nts). The

CAUH motif is highlighted by the black squares. **d** Enrichment analysis of the CAUH motif within the iCLIP CITS identified for control (scr) or *RPSAP52*-depleted cells (sh4). **e** Western Blot to assess HMGA2 protein levels in control (scr) or *RPSAP52*-depleted (sh4 and sh1) A673 cells. **f** UCSC Genome Browser view of *HMGA1*, *IGF1R* and *NRAS* 3'UTR with the read coverage from IGF2BP2 iCLIP experiment. Results from control (scr) or *RPSAP52* (sh4) samples are shown. The position of statistically significant CITS and peaks are indicated. **g** RNA stability of IGF2BP2 targets upon *RPSAP52* knockdown. A673 control (scr) or *RPSAP52*-depleted cells (sh4 B11) were treated with 5µg/ml Actinomycin D or DMSO for the times indicated before harvesting, as indicated in the drawing. mRNA levels for each gene at each time-point were then assessed by RT-qPCR (graphs). Data are means ±SD, and error bars represent data from 3 independent Actinomycin D treatments. **h** Western Blot analysis of IGF2BP2 protein levels upon LIN28B depletion. Both control and *RPSAP52*-depleted A673 cells were subject to LIN28B depletion by means of siRNAs, as indicated. **i**, IGF2BP2 immunoprecipitation from the cells in (**h**) followed by Western Blot to assess IGF2BP2 pull-down. **j** RNA from 90% of the pull-down in (**i**) was extracted and analyzed by RT-qPCR. Identity of the genes analyzed are indicated in each graph. Data are means ±SD, and error bars represent the results from 4 replicates of RT-qPCR analysis (*$P<0.05$, ns=not significant, two-tailed student *t*-test). Source data for **g** and **j** are in Oliveira-Mateos et al_Source Data 1. Unprocessed scans are available in Oliveira-Mateos et al_Source Data 2.

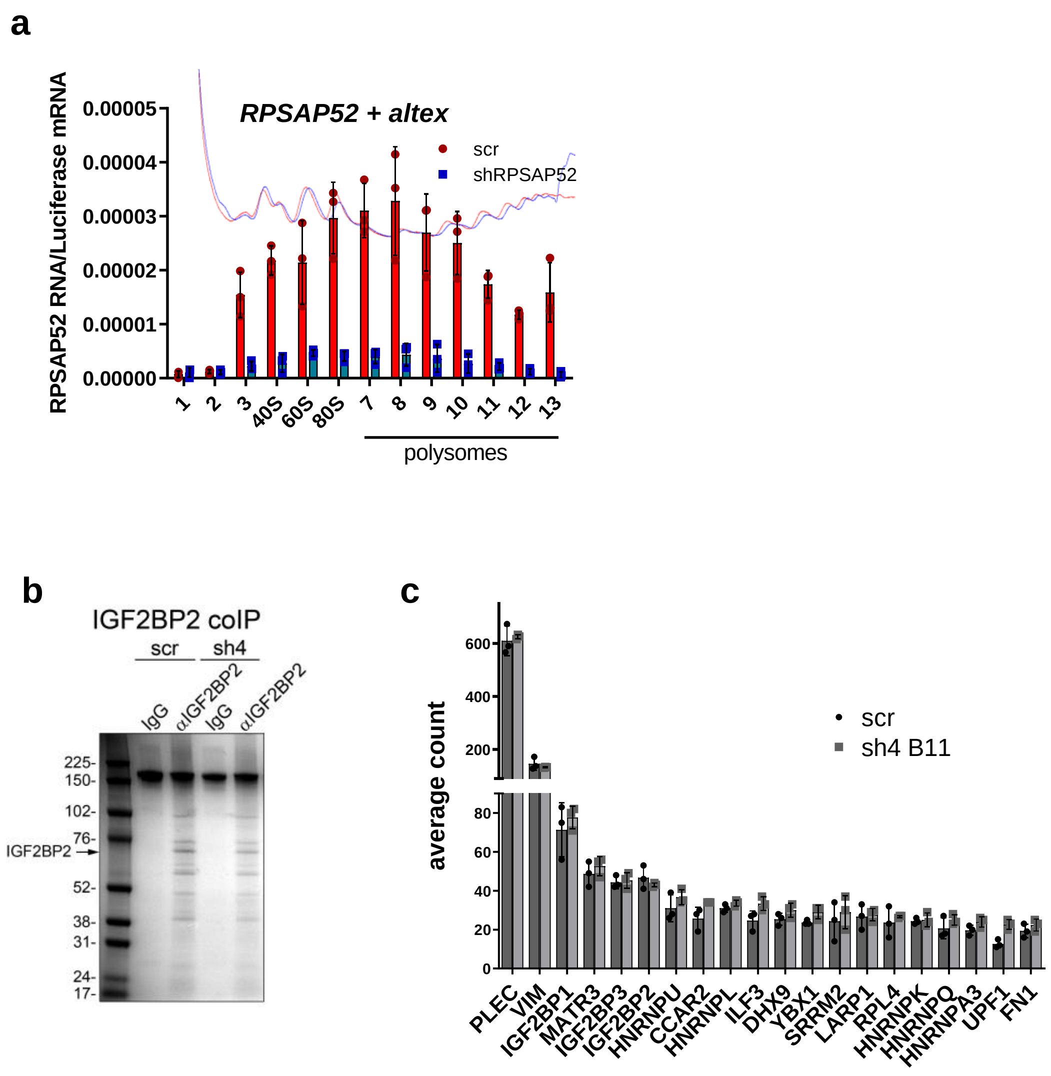

Supplementary Fig. 6. *RPSAP52* depletion does not change neither IGF2BP2 affinity for protein binding partners nor global translation efficiency. a *RPSAP52 + altex* distribution across a polysome gradient in control (scr) or depleted (shRPSAP52) A673 cells. The presence of RNA in each fraction was analyzed by RT-qPCR. Data are means ±SD, and error bars represent the results from 3 replicates of the RT-qPCR reaction. The red and blue lines indicate absorbance at 260 nm for each fraction in control or depleted cells, respectively. **b** Coomassie staining of a IGF2BP2 coimmunoprecipitation experiment in control and *RPSAP52*-depleted A673 cells (sh4). Mouse IgG was used as a negative control. **c** Average counts from the peptides eluted in 3 IGF2BP2 coimmunoprecitation experiments. The first top 20 proteins with highest counts are represented. No statistical differences between conditions were found among the interactors with highest counts (BFDR=1 in all cases). Data are means ±SD. Source data for **a** and **c** are in Oliveira-Mateos et al_Source Data 1. Unprocessed scans are available in Oliveira-Mateos et al_Source Data 2.

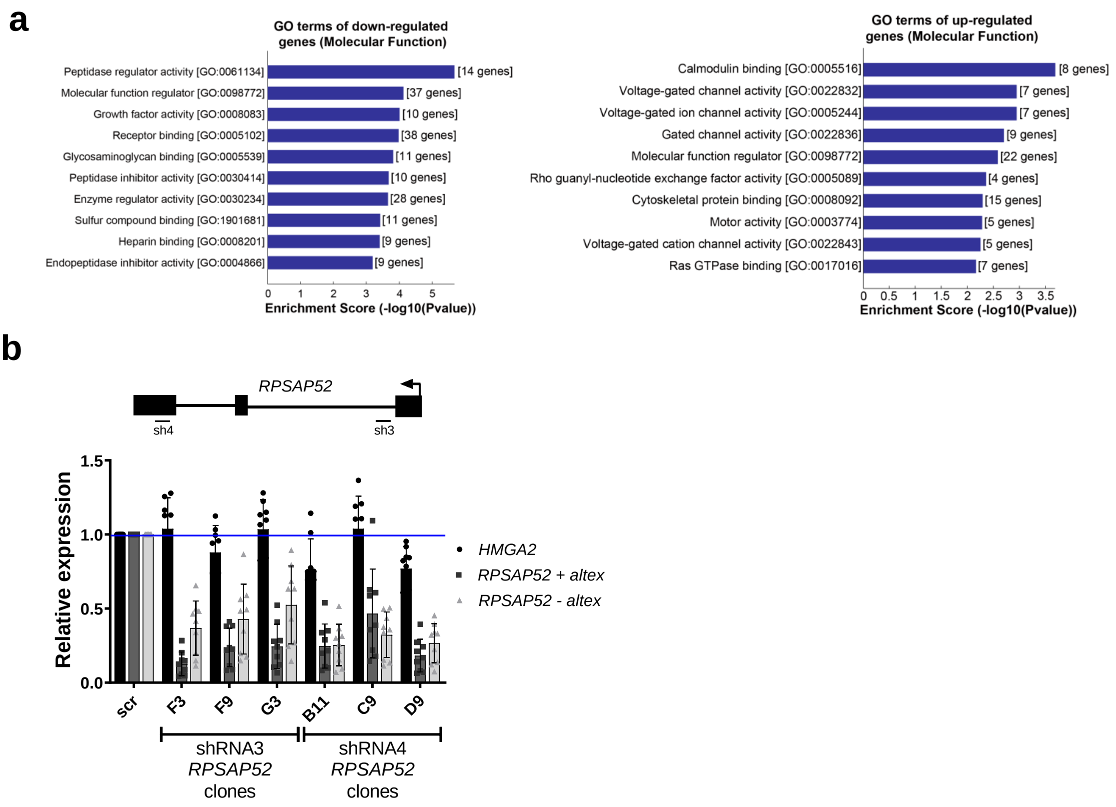

Supplementary Fig. 7. Impact of *RPSAP52* and LIN28B depletion in A673 cells. a Enriched GO terms for shRNA-*RPSAP52*-affected genes (two-tailed Fisher's exact test). The *y* axis shows Molecular Function terms and the *x* axis shows statistical significance. Enrichments for the down-regulated (left) or up-regulated (right) genes are shown. **b** *HMGA2* levels are not quantitatively altered upon *RPSAP52* knockdown with the sh3 and sh4 shRNA sequences. Location of the targeted regions on *RPSAP52* gene is indicated in the upper drawing. RT-qPCR assessment of *HMGA2* mRNA and *RPSAP52* transcripts is shown below. Data are means ±SD from 3 RT-qPCR replicates. Source data for **b** are in Oliveira-Mateos et al_Source Data 1.

Supplementary Table 1. Oligos used in this work

Name	Sequence 5' - 3'	Experiment
HMGA2for	CCTAAGAGACCCAGGGGAAG	RT-qPCR/RT-PCR
HMGA2rev	TCCAGTGGCTTCTGCTTTCT	RT-qPCR/RT-PCR
RPSAP52for	GAGCAAACACATCGGAGACA	RT-qPCR/RT-PCR
RPSAP52rev	AATTGGATTCCCACTGCAAG	RT-PCR
RPSAP+altexrev	CAGCTCGTTGCTCTCTGCTA	RT-qPCR
RPSAP52for2	ACTAGCACCAGTGGGCACAT	RT-qPCR
RPSAP-altexrev	CATGACAGGAATCTTTGAGTTAAG	RT-qPCR
GUSBfor	TGGTTGGAGAGCTCATTTGGA	RT-qPCR
GUSBrev	GCACTCTCGTCGGTGACTGTT	RT-qPCR
GAPDHfor	TCTTCTTTTGCGTCGCCAG	RT-PCR
GAPDHrev	AGCCCCAGCCTTCTCCA	RT-PCR
GAPDHfor2	TGCACCACCAACTGCTTAGC	RT-qPCR
GAPDHrev2	GGCATGGACTGTGGTCATGAG	RT-qPCR
RNU6Bfor	CTCGCTTCGGCAGCACA	RT-qPCR
RNU6Brev	AACGCTTCACGAATTTGCGT	RT-qPCR
c-FOSfor	CCGGGGATAGCCTCTCTTACT	RT-qPCR
c-FOSrev	CCAGGTCCGTGCAGAAGTC	RT-qPCR
IGF2BP2for	AGCCTGTCACCATCCATGC	RT-qPCR/RT-PCR
IGF2BP2rev	CTTCGGCTAGTTTGGTCTCATC	RT-qPCR/RT-PCR
NRASfor	ATGACTGAGTACAAACTGGTGGT	RT-qPCR/RT-PCR
NRASrev	CATGTATTGGTCTCTCATGGCAC	RT-qPCR/RT-PCR
IGF1Rfor	GGAATGAAGTCTGGCTCCG	RT-qPCR
IGF1Rrev	CAGCTGCTGATAGTCGTTGC	RT-qPCR
LIN28Afor	CTTTGTGCACCAGAGTAAGC	RT-qPCR
LIN28Arev	GACCCTTGGCTGACTTCTTA	RT-qPCR
LIN28Bfor	CATCTCCATGATAAACCGAGAGG	RT-qPCR/RT-PCR
LIN28Brev	GTTACCCGTATTGACTCAAGGC	RT-qPCR/RT-PCR
β-ACTINfor	CATCCGCAAAGACCTGTACG	RT-qPCR
β-ACTINrev	CCTGCTTGCTGATCCACATC	RT-qPCR
FLucfor	ACAGATGCACATATCGAGGTG	RT-qPCR
FLucrev	GATTTGTATTCAGCCCATATCG	RT-qPCR
TP53TG1for	CTTTCCTTTAATCTTCGGAGGC	RT-qPCR
TP53TG1rev	TGCCAGCTCTCAGAGTCCTT	RT-qPCR
MGST1for	ATTCATGGCTTTTGCATCC	RT-qPCR
MGST1rev	CTGCGTACACGTTCTACTCTGTC	RT-qPCR
CRABP1for	AAAACCTACTGGACCCGTGA	RT-qPCR
CRABP1rev	GAAAGTAGGAGCAAGCCAGC	RT-qPCR
CYR61for	AACGAGGACTGCAGCAAA	RT-qPCR
CYR61rev	CCCGTTTTGGTAGATTCTGG	RT-qPCR
CD109for	CCAAGATGCTTCAGTGTCC	RT-qPCR
CD109rev	CACAGGAGGACAGCTTCAC	RT-qPCR
PTPRZ1for	TACTGGCCAAATAAAGATGAGC	RT-qPCR
PTPRZ1rev	TGCCTCACTTCAAGTACATAATCA	RT-qPCR
STYK1for	CCTGTGGCTTTTTATCAGAGA	RT-qPCR
STYK1rev	AGGTCCCTAGGTGGAGGA	RT-qPCR
AREGfor	AGCCGACTATGACTACTCAGAAGA	RT-qPCR
AREGrev	CACTTTCCGTCTTGTTTTGG	RT-qPCR
CPT1Cfor	TCAAAGAGTTGCTGCCTGA	RT-qPCR
CPT1Crev	CAGCCGTGGTAGGACAGA	RT-qPCR
NPYfor	CCTCATCACCAGGCAGAG	RT-qPCR
NPYrev	TGGGAACATTTTCTGTGCTT	RT-qPCR
MTSS1for	ACCATCATCAGCGACATGA	RT-qPCR
MTSS1rev	GCCATGTCAGCCACTTTCT	RT-qPCR
TIAM1for	TGGAGGCAAAAGATTGTGTG	RT-qPCR
TIAM1rev	CCTCCTCCTCCCAAGAGACT	RT-qPCR
MICBfor	AAGAAAACATCAGCGGCAG	RT-qPCR
MICBrev	CATCCCTGTGGTCTCCTGT	RT-qPCR
RPSAfor	TCATTTCCTGCCGCCTGT	RT-qPCR
RPSArev	CATCCTCCTCCTTCATTTGC	RT-qPCR
RPSAP9for	ACCCCAATCCATTTTTACCC	RT-qPCR
RPSAP9rev	GGTCTTTTTGTGGCTTGATAGC	RT-qPCR
RPSAP58for	TCTGGAGCGAGAAAAAGAGC	RT-qPCR
RPSAP58rev	GGGTTCATCCACCATCTCAT	RT-qPCR
shRPSAP52-1for	gatccGTCCTTAAGCTCCTTGCAGTTTCAAGAGAACTGCAAGGAGCTTAAGGATTTTTTACGCGTg	Gene silencing
shRPSAP52-1rev	aattcACGCGTAAAAAATCCTTAAGCTCCTTGCAGTTCTCTTGAAACTGCAAGGAGCTTAAGGACg	Gene silencing
shRPSAP52-3for	gatccGTGCAAGACTCAGGAGCTATTCAAGAGATAGCTCCTGAGTCTTGCACTTTTTTACGCGTg	Gene silencing
shRPSAP52-3rev	aattcACGCGTAAAAAAGTGCAAGACTCAGGAGCTATCTCTTGAATAGCTCCTGAGTCTTGCACg	Gene silencing
shRPSAP52-4for	gatccGCACGGACTCTTAAGCAACATTCAAGAGATGTTGCTTAAGAGTCCGTGTTTTTTACGCGTg	Gene silencing
shRPSAP52-4rev	aattcACGCGTAAAAAACACGGACTCTTAAGCAACATCTCTTGAATGTTGCTTAAGAGTCCGTGCg	Gene silencing
bHMGA2for1	GGTAGTTTAAGTAATAGTAG	Methylation analysis
bHMGA2rev1	AAATAAACTAATACCCCCAC	Methylation analysis
bHMGA2for2	GTGGGGGTATTAGTTTATTT	Methylation analysis
bHMGA2rev2	ACCCCCAAAACTCTAACCCC	Methylation analysis
bHMGA2for3	GGGGTTAGAGTTTTGGGGGT	Methylation analysis
bHMGA2rev3	CAAACAAAACCCTCCACTCC	Methylation analysis
bHMGA2for4	GGAGTGGAGGGTTTTGTTTG	Methylation analysis
bHMGA2rev4	AAACTCAAAAACCTCTAAATC	Methylation analysis
bHMGA2for5	TAGAGGTTTTTGAGTTTTTT	Methylation analysis
bHMGA2rev5	ATTAACTTAAAACCCATAAA	Methylation analysis
bHMGA2for6	AATTAGTTTTATTTAATTAT	Methylation analysis
bHMGA2rev6	TAAAAAATTTTACTTAAATC	Methylation analysis
T7-RPSAP52for	GAAATTAATACGACTCACTATAGGGGCATCCCATTTAGAGAAT	*In vitro* biotin-transcription
RPSAP52-flrev	ATCGATCGCTCGAGTTTGCATCACAGAATTTT	*In vitro* biotin-transcription
T7-antiRPSAP52for	GAAATTAATACGACTCACTATAGGGTTTGCATCACAGAATTTT	*In vitro* biotin-transcription
RPSAP52-flfor	ATCGATCGCTCGAGGCATCCCATTTAGAGAAT	*In vitro* biotin-transcription
T7-RPSAP52altexfor	GAAATTAATACGACTCACTATAGGGAACTTGGGTGCTACCACTTGGATCC	*In vitro* biotin-transcription
RPSAP52dom1rev	CTTTAAGTCATGACAGGAATCT	*In vitro* biotin-transcription
T7-RPSAP52dom2for	GAAATTAATACGACTCACTATAGGGAGAAACTTTCACAATGTCTGG	*In vitro* biotin-transcription
RPSAP52dom2rev	GTGATGGCAATGTCCAATGG	*In vitro* biotin-transcription
T7-RPSAP52dom3for	GAAATTAATACGACTCACTATAGGGATGCAACAACAAGGGAGCTCCC	*In vitro* biotin-transcription
RPSAP52dom3rev	TTTGCATCACAGAATTTTATTTTTTA	*In vitro* biotin-transcription
T7-RPSAP52for-TnT	GAAATTAATACGACTCACTATAGGGaaggttccctgcaagcttct	TnT assay
RPSAP52rev-TnT	TTGCTTAAGAGTCCGTGCAA	TnT assay
