# Supplementary material for: The transcribed pseudogene RPSAP52 enhances the oncofetal HMGA2-IGF2BP2-RAS axis through LIN28B-dependent and independent let-7 inhibition
Source: Nat Commun. 2019 Sep 4;10:3979. doi: 10.1038/s41467-019-11910-6 (PMC6726650; doi:10.1038/s41467-019-11910-6)
Supplement: Supplementary file 3 — Description of Additional Supplementary Files [file 41467_2019_11910_MOESM3_ESM.pdf]

Description of Additional Supplementary Files

File Name: Supplementary Data 1
Description: This is an excel file with all significant peaks and CITS from the iCLIP-seq experiment in Figure 5.

File Name: Supplementary Data 2
Description: This is an excel file with all transcripts that are significantly altered in the expression arrays from Figure 7.
