# Supplementary material for: The transcribed pseudogene RPSAP52 enhances the oncofetal HMGA2-IGF2BP2-RAS axis through LIN28B-dependent and independent let-7 inhibition
Source: Nat Commun. 2019 Sep 4;10:3979. doi: 10.1038/s41467-019-11910-6 (PMC6726650; doi:10.1038/s41467-019-11910-6)
Supplement: Supplementary file 6 — Source Data [file 41467_2019_11910_MOESM6_ESM.zip › Source Data 2.pdf]

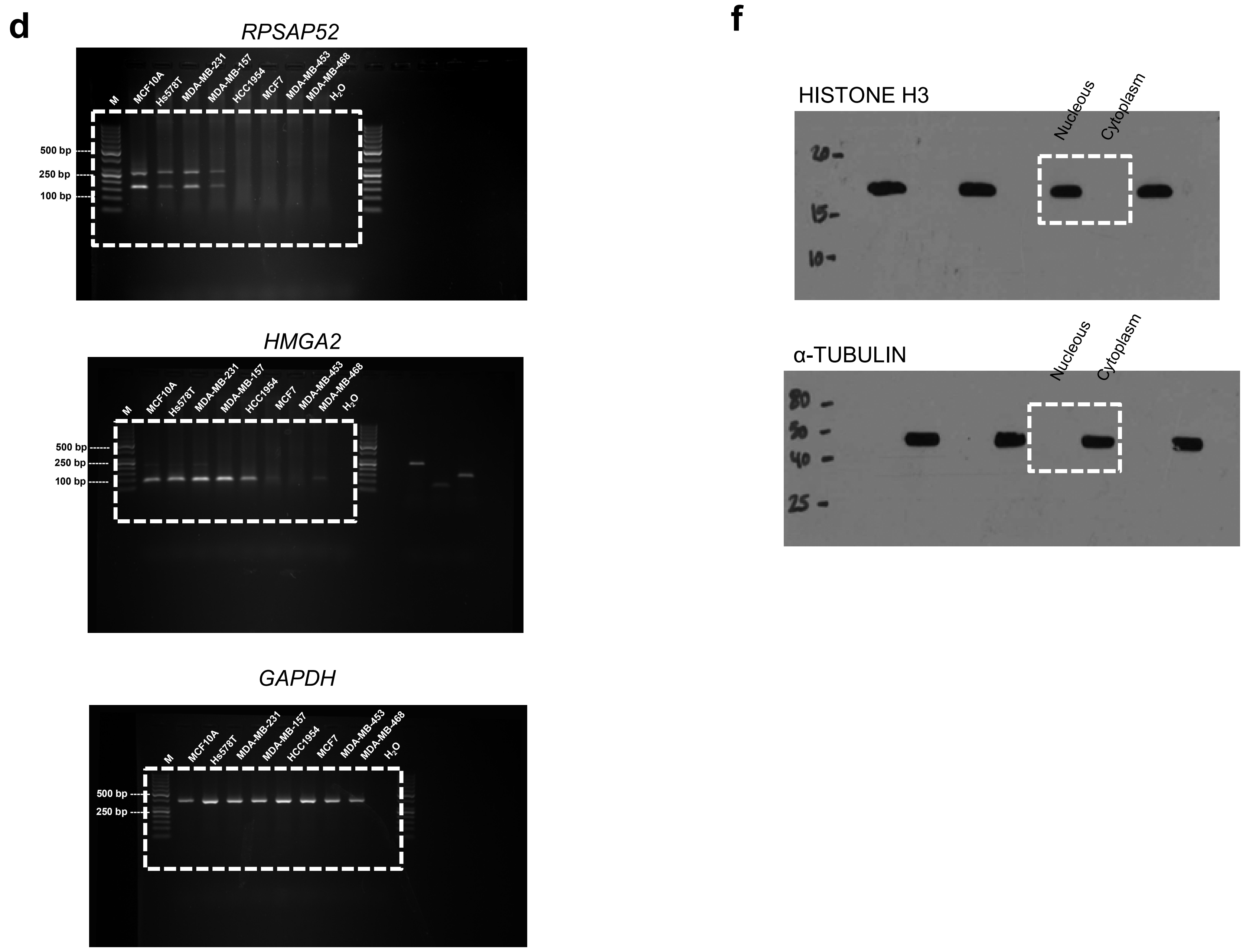

Uncropped images of PCRs and immunoblots displayed in the main **Figure 1**. Dashed boxes indicate the region that was cropped.

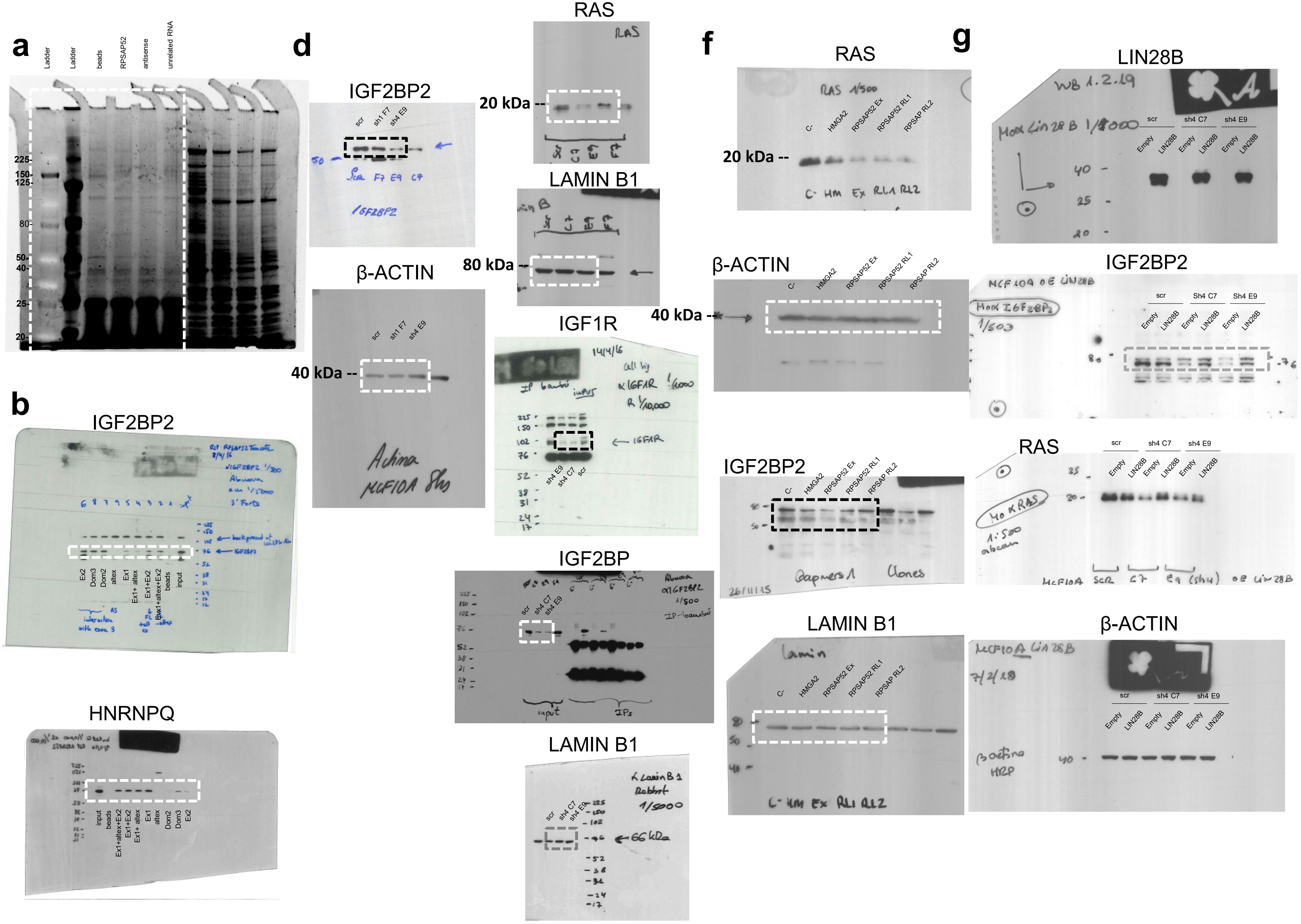

Uncropped images of immunoblots displayed in the main **Figure 2**. Dashed boxes indicate the region that was cropped.

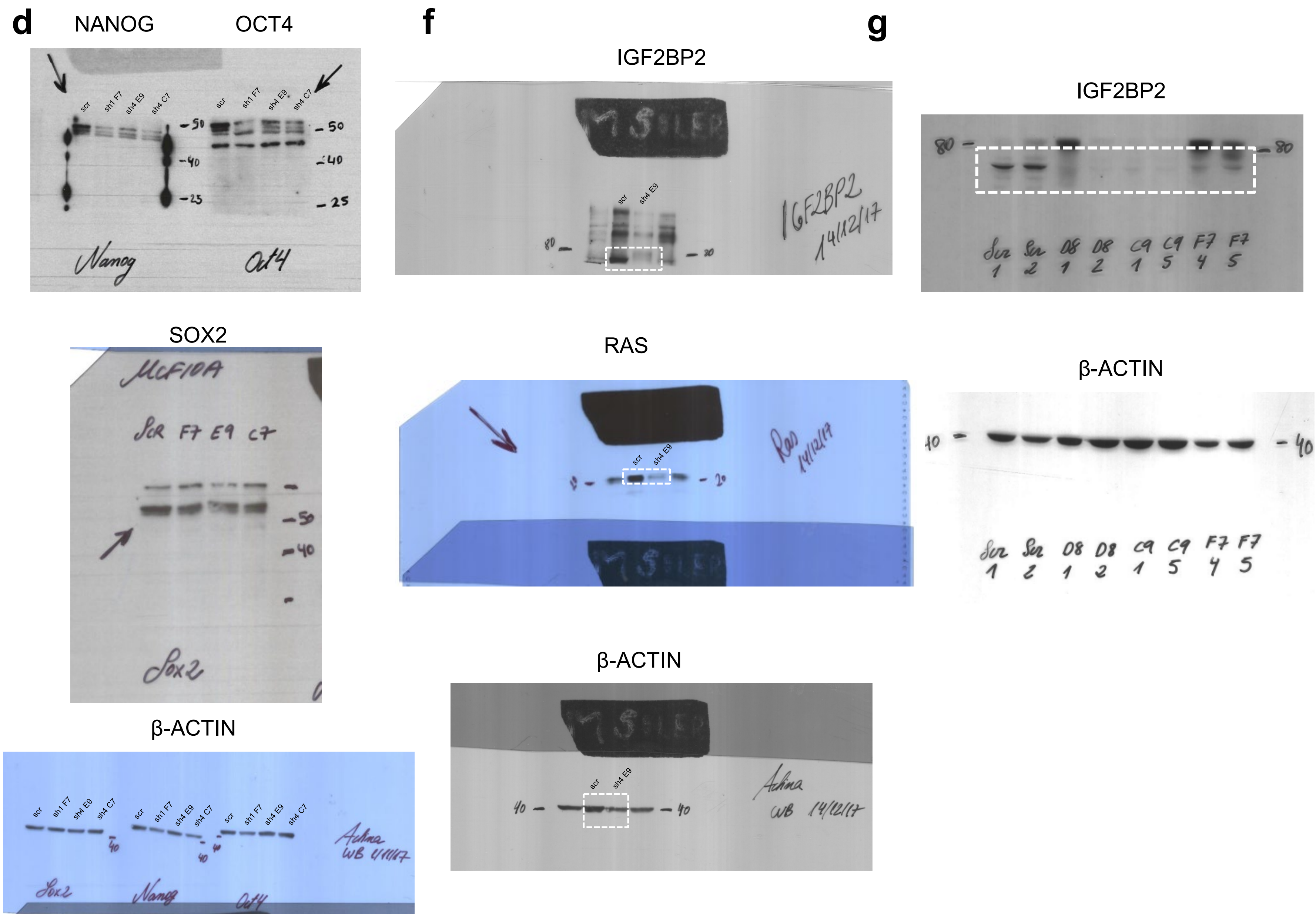

Uncropped images of immunoblots displayed in the main **Figure 3**. Dashed boxes indicate the region that was cropped.

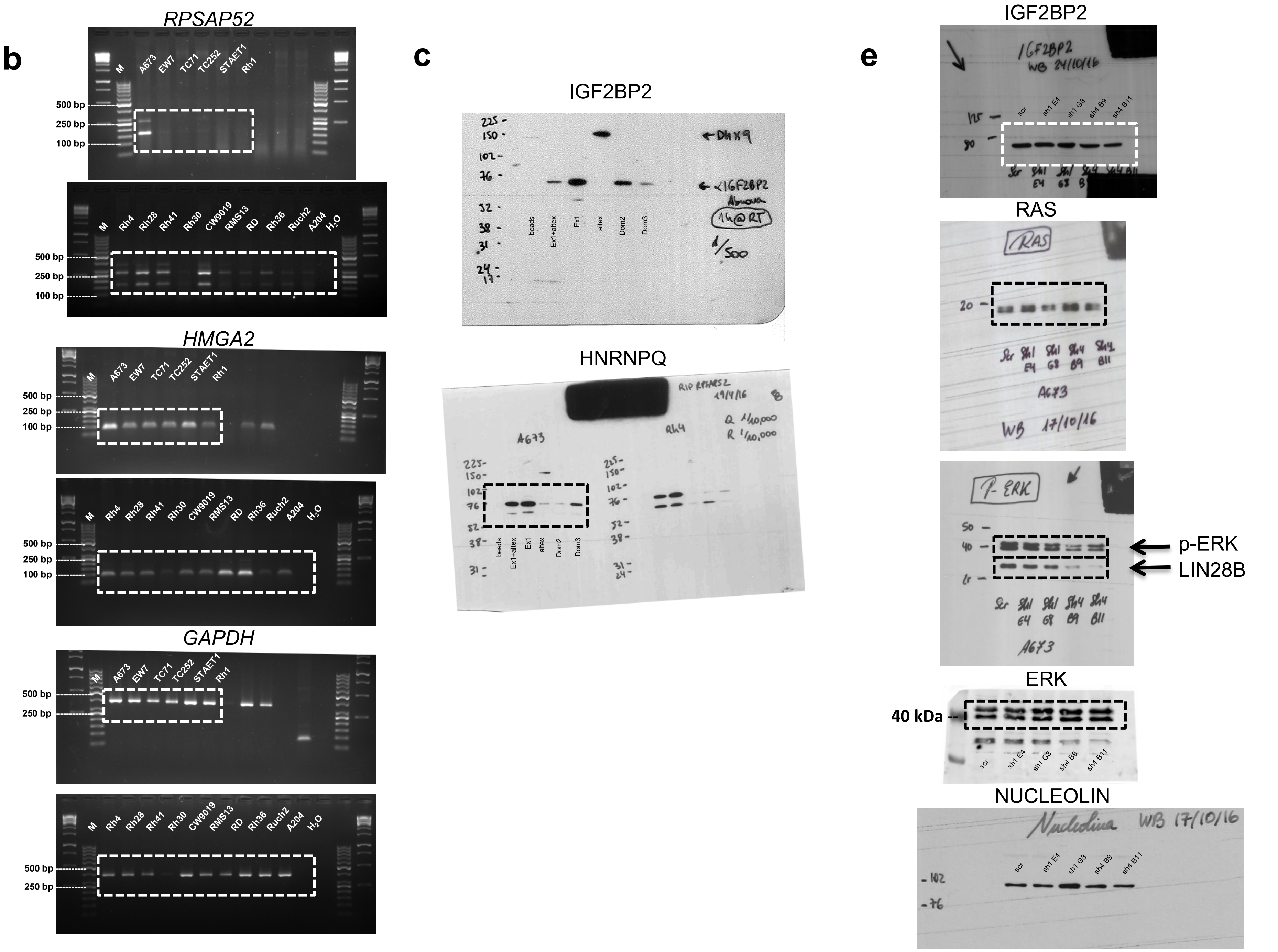

Uncropped images of PCRs and immunoblots displayed in the main **Figure 4**. Dashed boxes indicate the region that was cropped.

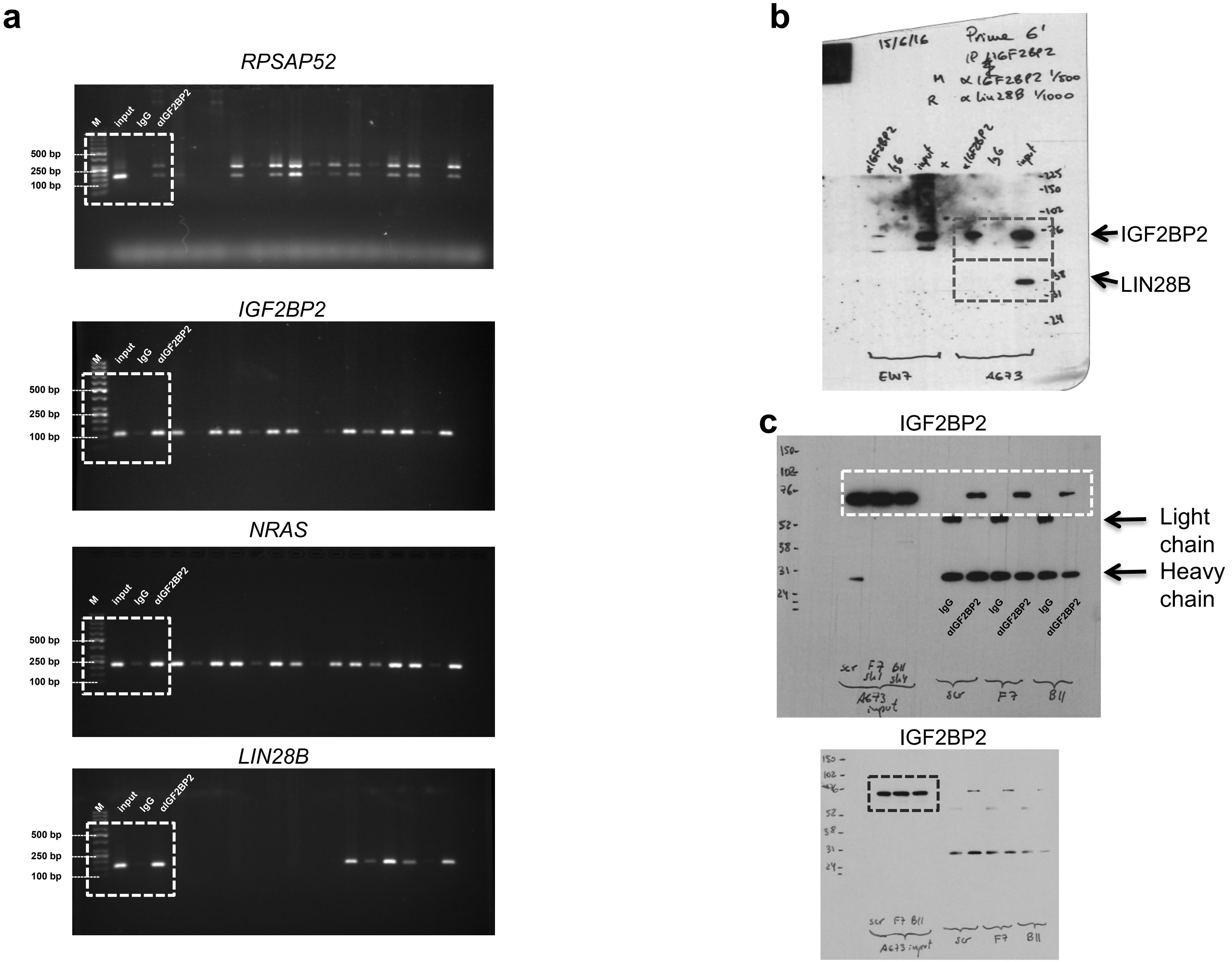

Uncropped images of PCRs and immunoblots displayed in the main **Figure 5**. Dashed boxes indicate the region that was cropped.

f

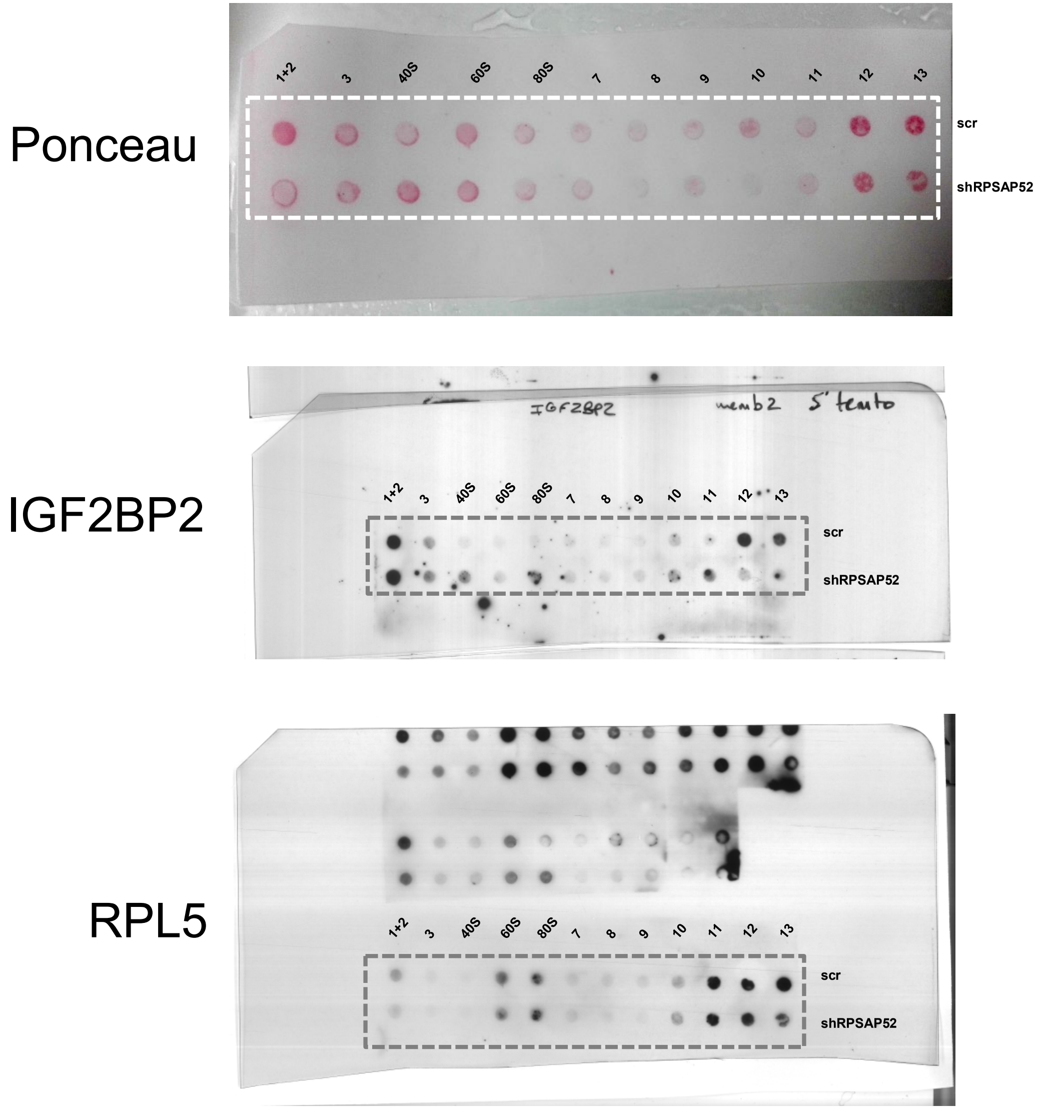

Uncropped images of immunoblots displayed in the main **Figure 6**. Dashed boxes indicate the region that was cropped.

a

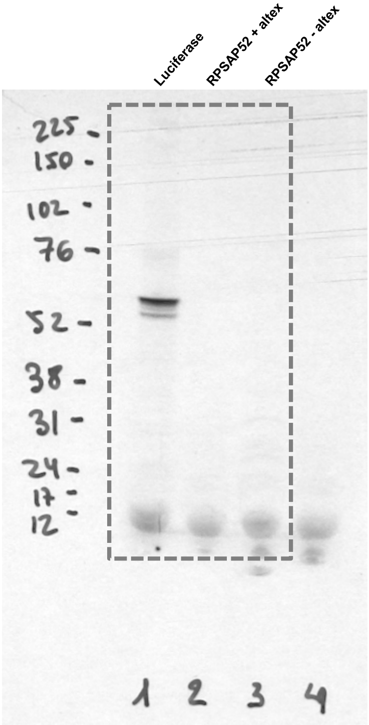

Uncropped images of immunoblots displayed in the **Supplementary Fig. 2a**. Dashed box indicates the region that was cropped.

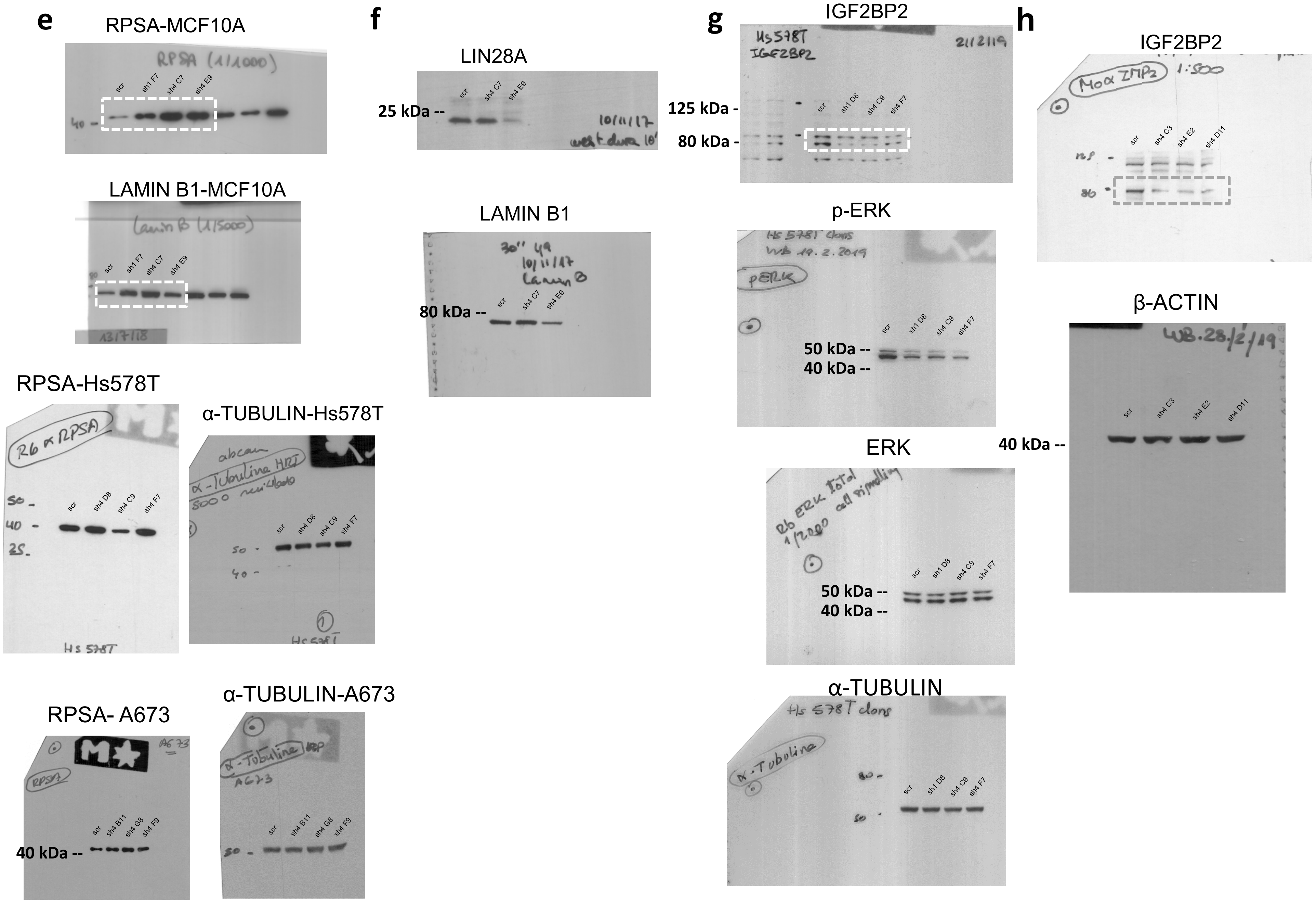

Uncropped images of immunoblots displayed in the **Supplementary Fig. 3**. Dashed boxes indicate regions that were cropped.

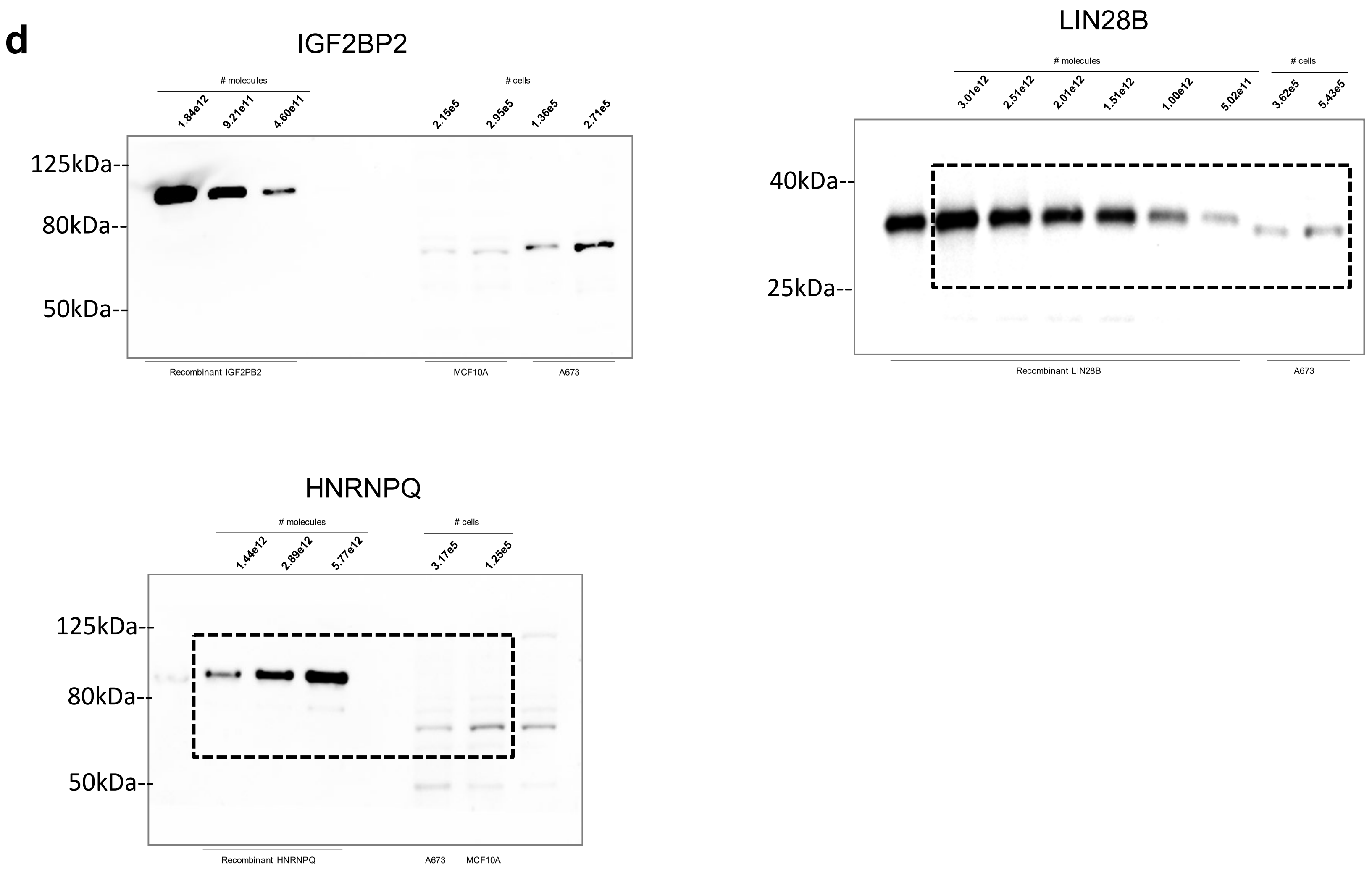

Uncropped images of immunoblots displayed in the **Supplementary Fig. 4**.

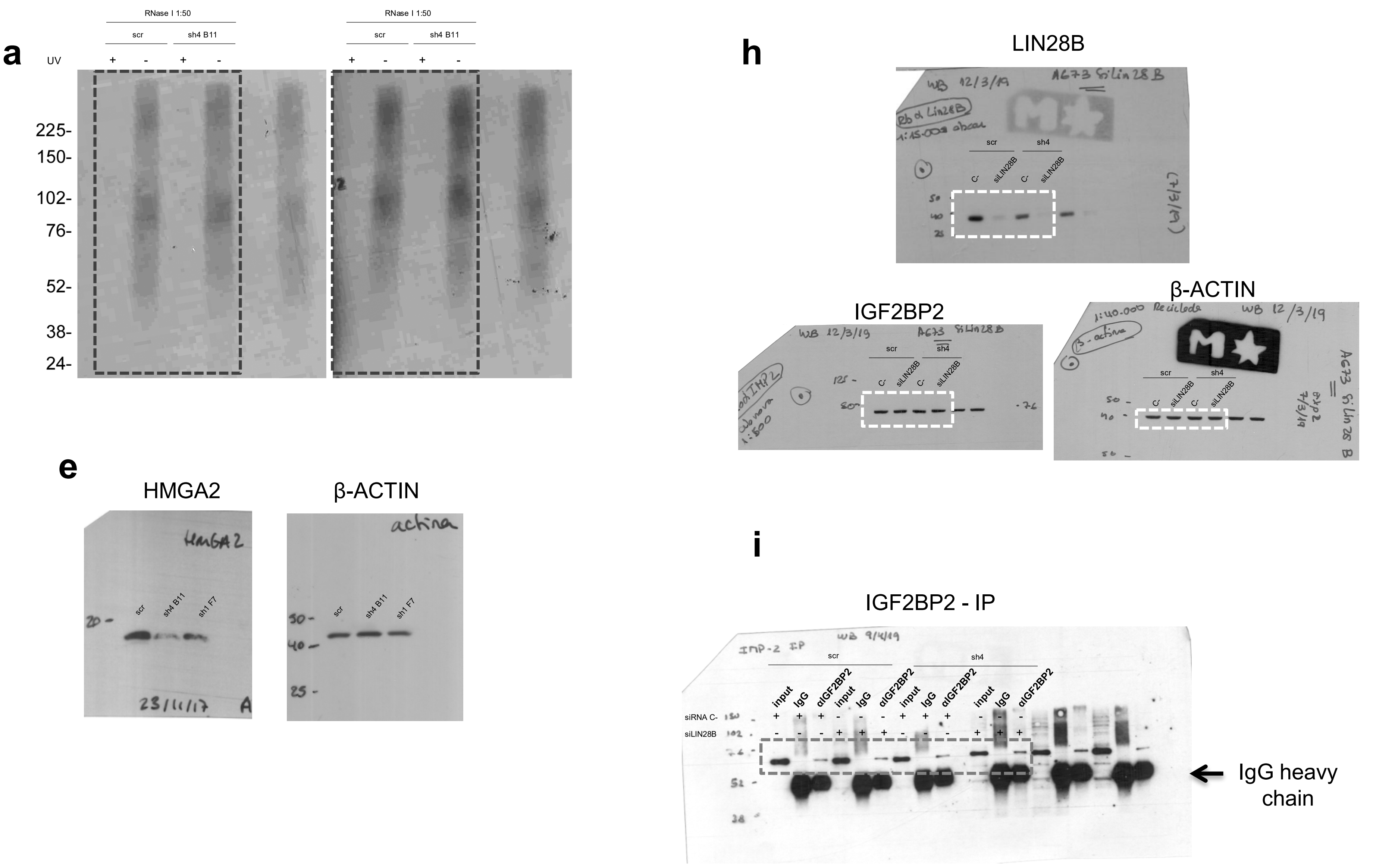

Uncropped images of autoradiographies and immunoblots displayed in the **Supplementary Fig. 5** .

b

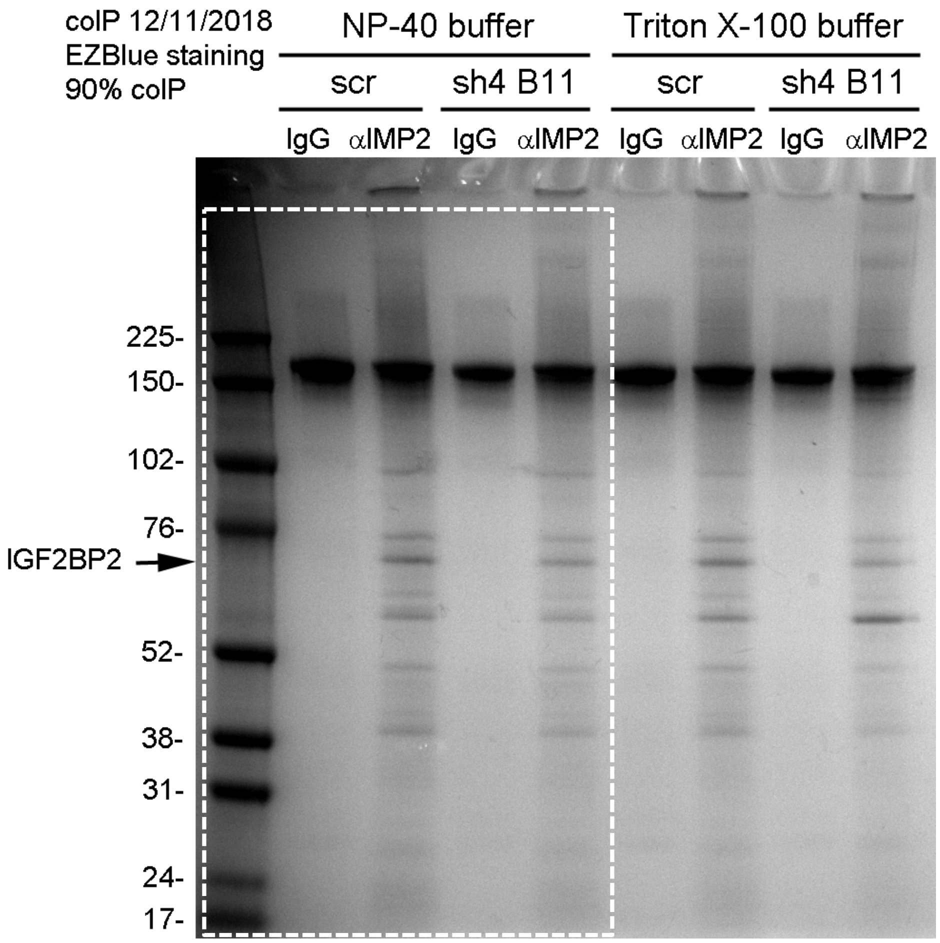

Uncropped image of Commassie staining displayed in the **Supplementary Fig. 6** .
